# A microfluidic device to acquire high-magnification microphotographs of yeast cells

**DOI:** 10.1186/1747-1028-4-5

**Published:** 2009-03-24

**Authors:** Shinsuke Ohnuki, Satoru Nogami, Yoshikazu Ohya

**Affiliations:** 1 Department of Integrated Biosciences, Graduate School of Frontier Sciences, University of Tokyo, Bldg. FSB-101, 5-1-5 Kashiwanoha, Kashiwa, Chiba 277-8562, Japan

## Abstract

**Background:**

Yeast cell morphology was investigated to reveal the molecular mechanisms of cell morphogenesis and to identify key factors of other processes such as cell cycle progression. We recently developed a semi-automatic image processing program called CalMorph, which allows us to quantitatively analyze yeast cell morphology with the 501 parameters as biological traits and uncover statistical relationships between cell morphological phenotypes and genotypes. However, the current semi-automatic method is not suitable for morphological analysis of large-scale yeast mutants for the reliable prediction of gene functions because of its low-throughput especially at the manual image-acquiring process.

**Results:**

In this study, we developed a microfluidic chip designed to acquire successive microscopic images of yeast cells suitable for CalMorph image analysis. With the microfluidic chip, the morphology of living cells and morphological changes that occur during the cell cycle were successfully characterized.

**Conclusion:**

The microfluidic chip enabled us to acquire the images faster than the conventional method. We speculate that the use of microfluidic chip is effective in acquiring images of large-scale for automated analysis of yeast strains.

## Background

Studies on cellular morphology have contributed to the discovery of factors involved in cell cycle control for various model organisms. In the yeast *Saccharomyces cerevisiae*, for example, many important findings related to cell cycle control have been reported, as yeast cell cycle progression is easily monitored via changes in cell morphology [1-3]. For growing yeasts, characteristic periodic morphological changes and structural rearrangements are observed morphologically such as bud emergence, bud formation, polarized actin localization, nuclear migration, karyokinesis, and cytokinesis [4-8]. During the G1 phase, yeast cell shape is a simple ellipsoid, and at the end of the G1 phase, the actin patches are localized at the presumed bud site [4]. When cells enter the S phase, DNA synthesis starts, and a bud emerges at the presumed bud site. During the S phase, the bud apically grows from the bud tip where actin patches are kept localized [4,5]. When the bud size becomes about two-thirds of that of mother, DNA synthesis ends and the bud is switched to isotropic growth with randomly redistributed actin patches in the bud [4,6,7]. Once the nucleus is localized at the neck and the actin patches are delocalized in the whole cell, the cell enters the M phase. During the M phase, the nucleus is divided to two nuclei and at the late M phase, the actin patches are localized again at the bud neck for cytokinesis [4,8]. A genetic approach for isolating and characterizing yeast temperature-sensitive mutants which accumulate at specific cell cycle stages upon temperature shifts (*cdc *mutants, originally reported in [9] and more than 60 genes are now annotated as *cdc *mutants on the *Saccharomyces *Genome Database [10]), led to the discovery of many key factors involved in cell cycle control [1]. In addition, cell morphology can be employed as the output of cell signaling because the accumulation of specific cell morphology is observed in response to extracellular stimuli such as mating pheromone [11]. However, the classification of cells of the comprehensive deletion mutant collection based on morphology was often subjective and time-consuming or was focused on limited information [12-15]. Conventional methods for classification are therefore unsuitable for conducting a detailed systematic comparative analysis using genomic tools including the yeast comprehensive deletion mutant collection.

We recently developed a high-throughput image processing program called CalMorph that lets us acquire high-resolution, quantitative information on cell morphology from fluorescent microscopic images of triple-stained (cell wall, actin and nuclear DNA) yeast cells [16]. We demonstrated that CalMorph is a powerful tool for studying cell cycle control, cell polarity, functional genomics, and comparative genomics [16-19]. We also demonstrated that the quantified morphological responses of mutants to stimuli let us characterize and predict gene functions [19].

Real-time observation of a living cell is required for studying the dynamics of cellular responses to extracellular stimuli. However, the current protocol for CalMorph image analysis is not suitable for characterizing the morphology of large-scale samples of living cells because the yeast cells must be fixed before staining. Thus, more high-throughput techniques for quantifying cell morphology at desired time points must be developed.

The aim of the present study is to develop the core device of a high-throughput system for acquiring images of living cells for CalMorph image analysis. We employed microfluidics using polydimethylsiloxane (PDMS), an optically transparent, soft elastomer suitable for observation with microscope and control of cells in channels [20-22]. Although many microfluidic devices have been developed for fluorescent microscopic imaging designed to measure the gene expression levels and the concentration of intracellular molecules, the device which was designed to quantify and analyze the cell morphology has never been reported [23-25]. We developed a microfluidic chip that holds cells in a desired orientation, which allows us to observe yeast cell morphology under high-magnification microscopy and acquire images of living yeast cells rapidly. Because the microfluidic chip is assumed to facilitate genome-wide phenotypic surveys through a combination with other microfluidic components, our device contributes not only to genomics and phenomics but also to the framework of the Micro Total Analysis System (μTAS) [26,27].

## Results

### Comparison of microfluidic chip method with conventional method

**Figure 1:**
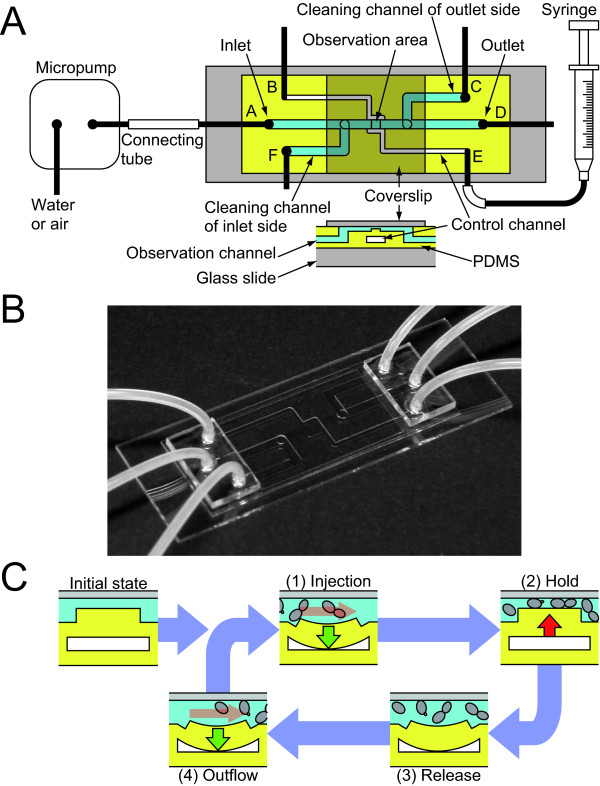
**Schematic illustration of the microfluidic device**. A. Overview and device settings. The top view of the device (upper side) and the cross section at the observation area (lower side) are illustrated. Each port from A to F was attached with a 2-mm silicone tube (black bar). Gray, yellow, and dark yellow areas indicate glass, PDMS, and the coverslip on the PDMS, respectively. Channels of white and sky-blue on the PDMS indicate the path of the air and the sample liquid, respectively. B. Photograph of the microfluidic device. C. Workflow of the chip. The illustrations indicate the cross section at the observation area of the chip. Vertical red and green arrows indicate the direction of the PDMS movement when directly and indirectly pressed by air, respectively. The horizontal red arrows in (1) and (4) indicate the flow direction of the sample liquid pressed by air. The observation of yeast cells is achieved with four cycle steps: (1) Injection, (2) Hold, (3) Release, and (4) Outflow. Yeast cells are softly held with PDMS pushed up by air pressure.

To improve the throughput of the image acquisition, we developed a two-layer microfluidic chip, and we compared the analyzed results obtained from images acquired by using the microfluidic chip and the conventional glass slide. Since the microfluidic chip was designed to retain the yeast cells beneath the coverslip by controlling the air pressure of the lower air-filled channel, it allowed us to acquire high-resolution cell images and enabled us to flush the cells on the chip quickly after image acquisition, contributing to the throughput (Figure 1, see Methods). The wild-type cells were used to compare the two methods, the microfluidic chip method and the conventional glass-slide method.

First, we compared the orientation of the cells. Because clear bud images are required for proper imaging, cells must be placed in the proper orientation so that the mother-bud cell axis is parallel to the X-Y imaging plane and the axes of each cell are in the same focal plane. Thus we calculated the proportion of the number of the budded cells placed in the proper orientation to the number of the budded cells and compared the results of two methods. To judge the orientation of bud, we employed a standard approach, serial section images of the same field, which is a standard approach but not suitable for high-throughput imaging. The cells were stained with Alexa488-ConA and five serial-section images (1 μm increment) of more than 200 cells were acquired. We then calculated the proportion of budded cells judged from the middle image of five focal planes to the budded cells judged from the all serial-section images by eye. Of 148 budded cells judged using all serial section images acquired by conventional glass slide method, 126 cells were correctly judged as budded cells from the middle image of five focal planes, indicating 85.1% of cells (95% confidence interval; 78.3%–90.4%) were in the desired orientation (bud and mother on the same focal plane). When we used the microfluidic chip, 139 of 154 cells (90.3%, 95% confidence interval; 84.4%–94.4%) were in the desired orientation, suggesting the assertion that the microfluidic chip can hold cells in a suitable orientation as well as the glass slide.

We investigated whether the cell shape in the microfluidic chip was undesirably changed. Because cells were pushed up by the PDMS membrane with air pressure from the control channel while acquiring images, the cells were possibly flattened by the mechanical force of the microfluidic chip. To exclude this possibility, we acquired images of the triple-stained cells on the microfluidic chip and the glass slide, quantified the morphology of the cell images with 501 parameters based on the cell shape, the nuclear shape and the position of actin patches by CalMorph, and statistically compared the results of two conditions. Of the 501 parameters, only 2 (DCV14-1_C, the coefficient of variation of nuclear size in mother: and DCV176_C, the coefficient of variation of nuclear long axis length in mother) were found to have values that were different between the two conditions by Mann-Whitney U-test at P < 0.01 (n = 5), but the false discovery rate (FDR) estimated that these two detections were expected to be false positives [28]. Therefore, no significant morphological differences between the microfluidic chip and conventional glass slide were found, supporting the compatibility of the two methods for quantitative analysis of yeast cell morphology.

We compared the image acquisition speed. The phase-contrast images were used for comparison because of the simplicity of the experimental condition. With the microfluidic chip, the average image acquisition speed of the phase contrast images for over 200 cells was 7.68 ± 1.02 images/min (n = 3); this was 2.62 times faster than the conventional method, which was 2.92 ± 0.04 images/min (n = 3) for more than 200 cells.

### Characterization of living cell morphology

We took advantage of the new microfluidic chip which had the capability to directly observe living cells in the medium. In order to quantify the living cell morphology with the phase-contrast image, the images were required to be processed to extract the cell outline before CalMorph image analysis because CalMorph was designed to process the fluorescent images. To characterize the cell shape of non-stained living cells, we developed a java-based program to preprocess the phase-contrast image before applying CalMorph (see Methods section). To validate the preprocessing program, we acquired both the phase-contrast images and FITC-ConA-stained image of fixed wild-type cells on the glass slide, calculated the values of 31 parameters out of 33 parameters by analyzing these images with CalMorph (33 parameters were a set of output from CalMorph if cell shape images were the only input, see Methods), and compared 31 parameter values between the two results. We detected no significant differences among the 31 parameters based on the Mann-Whitney U-test at P < 0.05 (n = 5), suggesting that the preprocessing did not significantly alter the output of CalMorph image analysis.

**Figure 2:**
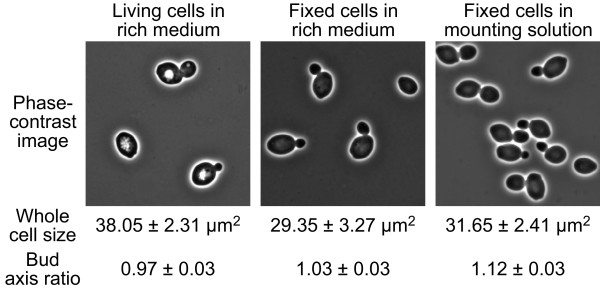
**Morphological characterization of the yeast cells from phase-contrast images**. Mean ± SD (n = 5) of whole cell size and bud axis ratios (the ratio of long axis length of bud to short axis length of bud) that reflects roundness of bud in each sample were calculated based on CalMorph output values C101 (whole cell size) and C114 (bud axis ratio), respectively.

To characterize the living cells and make comparisons with the conventional glass slide method, we acquired the phase-contrast images under three conditions: (i) living cells in the rich media on the microfluidic chip, (ii) fixed cells in the rich media on the microfluidic chip, and (iii) fixed cells in the mounting solution on the glass slide. After quantifying the cell morphology of each sample by CalMorph with preprocessing, the differences between the two combinations [(i) and (ii), and (ii) and (iii)] for 31 parameters were assessed using the Mann-Whitney U-test at FDR = 0.05 (n = 5). Of the 31 parameters, 17 were different between conditions (i) and (ii), many of which were related to cell size. This indicates that the fixed cells were significantly smaller than the living cells (Figure 2, see "whole cell size" as a striking example). Next, six parameters were different between conditions (ii) and (iii), showing that in the mounting solution, the fixed cells were significantly elongated (Figure 2, bud axis ratio that reflects roundness of bud as a striking example). These results indicate that the cell fixation and cell suspension into the mounting solution caused deformation of the yeast cells and suggest that the living cell morphology was successfully quantified.

### Cell cycle-dependent morphological change

**Figure 3:**
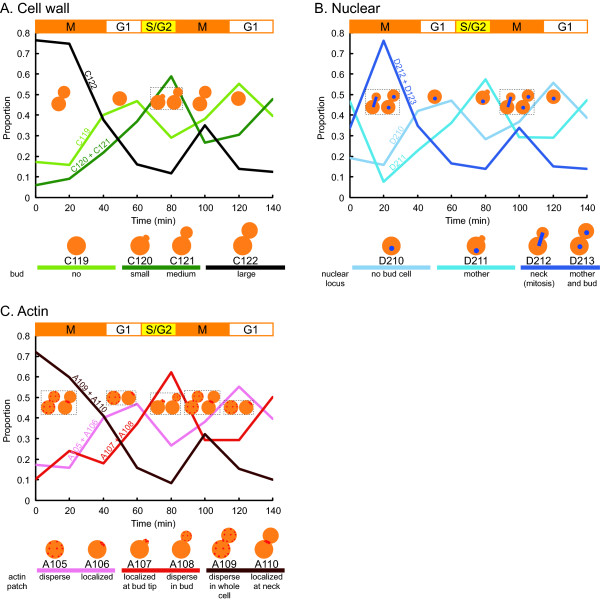
**Cell cycle-dependent changes in cell morphology**. Yeast cells were synchronized by nocodazole, released into the fresh rich media, and sampled every 20 min. The proportions of the cells showing the specific cell morphology at each time point calculated with CalMorph were plotted. Orange boxes on each figure indicate the approximate period of the cell cycle stage. A. Time-dependent changes in bud status. C119, C120, C121, and C122 indicate the proportion of unbudded cells, budded cells with a small bud, budded cells with a medium bud, and budded cells with a large bud, respectively. The schematic illustrations of the cell shape (orange) are depicted at the position on which the peak of each parameter value was observed. B. Time-dependent changes in nuclear status. D210, D211, D212, and D213 indicate the proportion of unbudded cells with one nucleus, budded cells with one nucleus in the mother, budded cells with one nucleus at the mother/bud neck, and budded cells with two nuclei (one each in the mother and bud), respectively. The schematic illustrations of the cell (orange) with a nucleus or nuclei (blue dots) are depicted at the position on which the peak of each parameter value was observed. C. Time-dependent changes in the actin status. A105, A106, A107, A108, A109, and A110 indicate the proportion of unbudded cells with dispersed actin patches, unbudded cells with localized actin patches, budded cells with localized actin patches at the bud tip, budded cells with dispersed actin patches in the bud, budded cells with dispersed actin patches in whole cell, and budded cells with localized actin patches at the mother/bud neck, respectively. Schematic illustrations of the cell (orange) with the actin patches (red dots) in the graph represent the peak position of the corresponding parameter value.

We examined whether the cell cycle can be monitored using the microfluidic chip, the preprocessing program, and CalMorph. To monitor cell cycle progression with the quantitative morphological data using the microfluidic chip, cells were synchronized in the M-phase with nocodazole treatment, periodically sampled and fixed after release to a normal cell cycle, and then double-stained cell images (DAPI and Rh-ph) in addition to the phase-contrast images were acquired (see Methods section). When we used the phase-contrast images, 12 parameters were discarded from the 501 parameters because they were calculated based on the fluorescent intensity of the cell wall images (see Methods). The rest of 489 parameter values out of 501 parameters were obtained by CalMorph image analysis with the preprocessing program (see Methods section). The time-dependent changes of 14 cell-cycle parameters are shown in Figure 3. The oscillations of parameter values were successfully observed and matched the expected cell cycle progression described before [4,5,7], demonstrating that the cell cycle progression can be monitored using our microfluidic device and software programs.

## Discussion

We developed a microfluidic chip that holds yeast cells in a desirable orientation on a single focal plane to continually acquire microscopic images for characterization of the cell morphology. Using the microfluidic chip, we could acquire images faster than the use of conventional glass slide. With the microfluidic chip, preprocessing program, and CalMorph, we successfully characterized live cell morphology and monitored cell cycle progression.

When we used the conventional glass-slide method, we had to be careful in selecting a visual field and adjusting focus, otherwise the images were not properly analyzed by CalMorph because of the undesirable orientation of cells. In contrary, with the microfluidic chip method, the clear images can be acquired without careful selection and focus adjustment because the microfluidic chip enables us to hold the cells into a single focal plane with the desirable orientation. We think that this contributes to accelerate the acquisition speed.

The microfluidic chip can be applied to the high-throughput microphotography system by automation. For the automation, the microfluidic chip control system is required. Since the fluid operation for the image acquisition is based on air-pressure, it is easy to develop a fluid control system driven by air-pump that is controlled by a computer [21-23]. If the microfluidic chip, microscope and image processing software programs are interconnected with each other, we can acquire images until desired number of cells has been analyzed without manual control.

The throughput of the system might be improved because the microscope is idle during three (injection, release and outflow) of four steps to acquire images. To minimize the idle time of microscope, fabricating the several sets of the microfluidic channels in parallel on a chip might be effective [20]. In addition, the redundancy of the parallel fabrication would be robust toward the accidents (ex. channel blocking). The parallelization promises the continuous running of the microscopic chip-scanning for long time, which would provide the genome-wide survey of the morphological phenotypes on various conditions in short period [26].

In large-scale experiments, the system combined with microplate to stock the input samples would be useful. Moreover, by combining other components such as microchemostat which is a miniaturized growth chamber on a chip [29], development of the micro total analysis system (μTAS: the system capable from the sampling to the detection on a chip [27]) which is capable from the cell culture to the phenotyping might be possible.

## Conclusion

We developed a microfluidic chip that can hold yeast cells in a desirable orientation so that we can continually acquire microscopic images of the cells to characterize the cell morphology. The advantage of the microfluidic chip is to facilitate fast image acquisition without careful image acquisition steps. We successfully characterized live cell morphology and monitored cell cycle progression with the microfluidic chip, preprocessing program, and CalMorph. Air pressure-based cell control and a small scale of microfluidic channels will be advantageous for automation and parallelization, accelerating genome-wide phenotypic surveys under various conditions.

## Methods

### Chip design, fabrication, and manipulation

The microfluidic chip has three kinds of microchannels: an observation channel for injecting the cell suspension and observing the cells, a control channel for controlling the depth of the observation channel, and a cleaning channel for cleaning the observation channel (Figure 1A). The microfluidic chip has a two-layer structure with the liquid-filled observation channel (upper layer) and the air-filled control channel (lower layer). From the top side, the two channels appear to cross at the observation area, but are actually separated by 100 μm of PDMS at the observation area. The depth of the observation channel at the observation area was manually controlled by the air pressure of the control channel supplied by a syringe [30]. We purchased the custom-designed chip (Fluidware Technologies Inc., Saitama, Japan) (Figure 1B).

The microfluidic chip was designed to run with four cycle steps (Figure 1C). Initially, distilled water (DW) flowed through from the inlet (port A) to the outlet (port D) using a micropump (SDMP302; Star Micronics, Shizuoka, Japan) with the micropump controller (MPC-200; Star Micronics) to wash the inside of the microchannel. Before injecting samples, ports B and C were closed, and ports D and F were opened (Figure 1A).

Step 1 (injection): To inject the cell suspension, the connecting tube was detached at the inlet side. A 30-μl sample of cell suspension (1 × 10^8 ^cells/ml) was then directly injected into the silicon tube connected to the inlet using a 200-μl micropipette and loaded to the observation channel of the inlet side. After closing port F and attaching the connecting tube to the inlet, the sample was gently loaded to the observation area by a micropump without any (positive and negative) pressure of the control channel.

Step 2 (hold): The inner air of the control channel was pressed by the syringe, and the cells in the observation channel were held by PDMS pushed up by the air pressure. During this step, the microscopic images were continually acquired by surveying the 2 × 2 mm observation area.

Step 3 (release): After image acquisition, port B was opened, and the syringe was returned to the initial state. The cells were released from the surface of the coverslip.

Step 4 (outflow): Port F was opened, and cells in the observation channel of the inlet side were flushed out by DW using the micropump. Port F was then closed, and the cells on the observation area were flushed out by DW.

When many cells were left in the observation channel, the cells were removed by ultrasonic treatment using a bath-type sonicator (model 2510J-MT; Branson Ultrasonic, CT, USA), filling the channels with the cleaning solution containing 0.1 M Tris-HCl (Sigma-Aldrich, MO, USA) at pH 7.5, 50 μg/ml Zymolyase 100T (Seikagaku Corporation, Tokyo, Japan), 2 μl/ml mercaptoethanol (Nacalai Tesque Inc., Kyoto, Japan), and 1% (v/v) Nonidet P-40 (Nacalai Tesque Inc.). After observation, the microfluidic chip was filled with 20% ethanol (Wako Pure Chemical Industries, Ltd., Osaka, Japan) and stored at 4°C.

### Yeast strain and image acquisition

Wild-type *S. cerevisiae *strain BY4743 was purchased from the European *Saccharomyces cerevisiae *Archive for Functional Analysis (EUROSCARF: ) and used in this study. The rich medium for growing *S. cerevisiae *was YPD medium that contained 1% (w/v) Bacto yeast extract (BD Biosciences, CA, USA), 2% (w/v) Bacto peptone (BD Biosciences), and 2% (w/v) glucose.

For live cell imaging, cells (8 × 10^6 ^cells) at the log phase in the rich media at 25°C were collected and resuspended to 1 × 10^8 ^cells/ml with the rich media. For conventional CalMorph imaging, cells were fixed in the rich media by adding 37% formaldehyde (Wako Pure Chemical Industries, Ltd.) and 1 M potassium phosphate buffer (pH 6.5) at a final concentration of 3.7% and 0.1 M, respectively. Conventional triple-staining of the yeast cells on the glass slide were performed as described previously [16]. In some experiments, Alexa Fluor 488-conjugated concanavalin A (Alexa488-ConA; Invitrogen, CA, USA) was used for fluorescent staining of mannoprotein (localized in the cell wall) instead of fluorescein isothiocyanate-conjugated ConA (FITC-ConA; Sigma-Aldrich) because Alexa488-ConA was brighter and more photostable than the FITC-ConA, preferable for observation on microfluidic device. For the fluorescent observation using Alexa488-ConA on the microfluidic chip, the cells were suspended into PBS buffer (Takara Bio Inc., Shiga, Japan) instead of the mounting solution containing 1 mg/ml *p*-phenylenediamine (Sigma-Aldrich), 9.975% (v/v) phosphate-buffered saline (PBS, Takara Bio Inc.), 0.025% (v/v) 0.1 N NaOH (Wako Pure Chemical Industries, Ltd.), and 90% (v/v) glycerol (Merck MGaA, Darmstadt, Germany).

### Quantification of cell morphology and statistical tests

Image analysis with CalMorph was performed as described previously [16]. CalMorph used in this study was version 1.3, which was an improved version of the originally described CalMorph (ver. 1.1) and was designed to characterize the diploid morphology. To use phase-contrast images as cell wall images for CalMorph analysis, we developed another java-based program. The program was a preprocessing program designed to extract the outline of cells and convert the phase contrast images to the CalMorph analyzable images. Thirty-three parameters from cell wall-stained images and 501 parameters from triple-stained images were available using CalMorph. We discarded parameters reflecting fluorescent intensity of cell wall staining for analysis of phase-contrast images. As the result, thirty-one parameters from phase-contrast images and 489 parameters from double-stained [4',6-diamidino-2-2-phenylindole (DAPI, Wako Pure Chemical Industries, Ltd.) and rhodamine-phalloidin (Rh-ph, Invitrogen)] images in addition to phase-contrast images were available. The software is available on request from the authors.

Statistical analysis of the quantified morphological data was performed using R . The differences in cell morphology under each condition were tested using the Mann-Whitney U-test with the false discovery rate (FDR) [28,31,32].

### Synchronized cell culture

Yeast cells were grown in the 60 ml rich media at 30°C using a 300-ml shaking flask. At the log-phase of 8 × 10^6 ^cells/ml, 600 μl of 15 mg/ml nocodazole (Sigma-Aldrich) in DMSO (Wako Pure Chemical Industries, Ltd.) was added (final 0.15 mg/ml of nocodazole), and cells were cultured for 3 h. M-phase arrested cells were washed twice with the rich media, resuspended with 60 ml of the fresh rich media, cultured at 30°C, sampled (1 ml) every 20 min, and fixed by adding 125 μl of 1 M potassium phosphate buffer (K-Pi buffer, pH 6.5) and 125 μl of 37% formaldehyde.

## Competing interests

The authors declare that they have no competing interests.

## Authors' contributions

SO coordinated the study, and carried out the experiments, the software development and the data analysis. SN supervised the experiments during the study. YO designed the study and wrote the manuscript. All authors read and approved the final manuscript.
